# Identification of Criticality in Neuronal Avalanches: I. A Theoretical Investigation of the Non-driven Case

**DOI:** 10.1186/2190-8567-3-5

**Published:** 2013-04-23

**Authors:** Timothy J Taylor, Caroline Hartley, Péter L Simon, Istvan Z Kiss, Luc Berthouze

**Affiliations:** 1 Centre for Computational Neuroscience and Robotics, University of Sussex, Falmer, Brighton, BN1 9QH, UK; 2 Institute of Child Health, University College London, London, WC1E 6BT, UK; 3 Centre for Mathematics and Physics in the Life Sciences and Experimental Biology, University College London, London, WC1E 6BT, UK; 4 Institute of Mathematics, Eötvös Loránd University Budapest, Budapest, Hungary; 5 School of Mathematical and Physical Sciences, Department of Mathematics, University of Sussex, Falmer, Brighton, BN1 9QH, UK

## Abstract

In this paper, we study a simple model of a purely excitatory neural network that, by construction, operates at a critical point. This model allows us to consider various markers of criticality and illustrate how they should perform in a finite-size system. By calculating the exact distribution of avalanche sizes, we are able to show that, over a limited range of avalanche sizes which we precisely identify, the distribution has scale free properties but is not a power law. This suggests that it would be inappropriate to dismiss a system as not being critical purely based on an inability to rigorously fit a power law distribution as has been recently advocated. In assessing whether a system, especially a finite-size one, is critical it is thus important to consider other possible markers. We illustrate one of these by showing the divergence of susceptibility as the critical point of the system is approached. Finally, we provide evidence that power laws may underlie other observables of the system that may be more amenable to robust experimental assessment.

## 1 Introduction

A number of in vitro and in vivo studies [1-4] have shown neuronal avalanches—cascades of neuronal firing—that may exhibit power law statistics in the relationship between avalanche size and occurrence. This has been used as prima facie evidence that the brain may be operating near, or at, criticality [5,6]. In turn, these results have generated considerable interest because a brain at or near criticality would have maximum dynamic range [7-10] enabling it to optimally react and adapt to the dynamics of the surrounding environment [5,11] whilst maintaining balanced neuronal activity [12-14]. Neuropathological states (e.g., epileptic seizures) could then be conceptualised as a breakdown of, or deviation from, the critical state; see [15], for example. Furthermore, these findings have led to the notion that the brain may self-organise to a critical state [16], i.e., its dynamics would be driven toward the critical regime by some intrinsic mechanism and not be dependent on external tuning. In support of this view, Levina and colleagues [17] showed analytically and numerically that activity-dependent depressive synapses could lead to parameter-independent criticality. 

The interpretation that neuronal activity is poised at a critical state appears to be mostly phenomenological whereby an analogy has been developed between the propagation of spikes in a neuronal network and models of percolation dynamics [18] or branching processes [19,20]. Remarkable qualitative similarities between the statistical properties of neuronal activity and the above models have given credence to this analogy, however, the question remains as to whether it is justified. Indeed, various key assumptions underlying percolation dynamics and branching processes are typically violated in the neuroscience domain. For example, full sampling, which is required in order to assess *self-organised* criticality, is unattainable even in the most simple in vitro scenario and yet it has been shown that sub-sampling can have profound effects on the distribution of the resulting observations [21]. On a related note, and more generally, the formal definition of a critical system as one operating at, or near, a second-order (continuous) phase transition is problematic since the concept of phase transition applies to systems with infinite degrees of freedom [22]. Many neuroscience authors address this by appealing to the concept of finite size scaling and many published reports implicitly assume that distributions are power law with truncation to account for the so-called finite size effect. Typically, such reports adopt an approach whereby (a) scale invariance is assessed through finite size scaling analysis, confirming that upon rescaling the event size, the curves collapse to a power law with truncation at system size (but see below regarding the definition of system size); (b) the parameters of statistical models are estimated, typically over a range of event size values that are rarely justified; and (c) the best model is determined by model selection, in which power law and exponentially truncated power law are compared to alternatives such as exponential, lognormal and gamma distributions; see [23] for a typical example. Whilst greater rigour in the statistical treatment of the assessment of the presence of power laws has been attained following Clauset and colleagues’ influential paper [24], what seems to be lacking is a rigorous treatment as to why a power law should be assumed to begin with. Although this question is particularly pertinent to the neurosciences, it should be noted that similar questions remain open in the field of percolation theory (e.g., [25,26]), namely: (i) how does the critical transition behaviour emerge from the behaviour of large finite systems and what are the features of the transition? (ii) what is the location of the scaling window in which to determine the critical parameters? 

This paper specifically seeks to address the following questions: 

1. Assuming that the whole brain, or indeed a region of interest defined by where data can be obtained, is operating at criticality, can we reasonably expect power law statistics in neural data coming from a very small (possibly sub-sampled) subset of the system? If not, what would be the expected distribution? Sornette [27] states that the Gamma distribution is “found in critical phenomena in the presence of a finite size effect or at a finite distance from the critical point.” Jensen [28] claims that finite-size systems often show an exponential cut-off below the system size. However, we are not aware of any study in which the distribution of event sizes in a finite-size system set to operate at a critical regime has been investigated. 

2. In a finite-size system, is it reasonable/possible to perform a robust statistical assessment of power law statistics? Even the application of a rigorous model selection approach will lead to different results depending on the choice of the range of event sizes and the number of samples being considered [29]. The issue of range selection is of particular interest. Whilst the notion of system size is clear in models of criticality such as the Abelian sandpile where (i) there is full sampling, (ii) the number of sites is finite, and (iii) there is dissipation at the edges, system size is much less obvious where re-entrant connections exist, making it possible, in principle, for avalanches to be of infinite size. Here, the counting measure which leads to the definition of an avalanche is important. Counting the number of neurones involved in an avalanche will lead to a clearly defined system size, whereas counting the total number of activations—the de facto standard, e.g., [12,17,30]—will not. Furthermore, it should also be noted that the presence of re-entrant connections invalidates the standard theory of branching processes [20], and makes a rigorous determination of the branching parameter *σ* problematic if not impossible, e.g., in the presence of avalanches merging.

3. Are there other markers of criticality that may be more amenable to characterisation and that should be considered instead of, or in addition to, the statistics of event sizes? The need for such markers in neuroscience has been recognised (see [29] for example) and a number of studies have investigated long-range temporal correlations (power-law decay of the autocorrelation function) in amplitude fluctuations [31] and in inter-burst intervals [32,33]. However, a theoretical account of how those may relate to one another is lacking (although see the recent work in [34]). Other markers of criticality (or markers of transitions) have been associated with critical physical systems, e.g., divergence of susceptibility and slowing of the recovery from perturbations near the critical point [27], however, we are not aware of any theoretical or empirical study investigating them in a neuroscience context. 

One way to address these questions more rigorously is to use simplified, but therefore more tractable conceptual models (e.g., [35]). In this paper, we use a model of a purely excitatory neuronal system with simple stochastic neuronal dynamics that can be tuned to operate at, or near, a second-order phase transition (specifically, a transcritical bifurcation). The simplicity of the model allows us to analytically calculate the exact distribution of avalanche sizes, which we confirm through simulations of the system’s dynamics. We study our model at the critical point and compare our exact distribution to the explicit but approximate solution proposed by Kessler [36] in an analogous problem of modelling disease dynamics. We confirm that Kessler’s approximate solution converges to our exact result. Importantly, we show that, in the proposed finite-size system, this distribution is not a power law, thus highlighting the necessity of considering other markers of criticality. We thus analyse two potential markers of criticality: (i) the divergence of susceptibility that arises in the model as we approach the critical point, (ii) the slowing down of the recovery time from small disturbances as the system approaches the critical point. Finally, we speculate on a sufficient but not necessary condition under which our exact distribution could converge to a true power law in the limit of the system size. 

## 2 The Stochastic Model

We start from the stochastic model of Benayoun et al. [12], which we simplify to the most trivial of models. A fully connected network of *N* neurones is considered with purely excitatory connections (as opposed to the excitatory and inhibitory networks considered in [12]). Within the network, neurones are considered to be either quiescent (Q) or active (A). Taking a small time step *dt* and allowing dt→0 the transition probabilities between the two states are then given by:

$$\begin{array}{rl} P(Q\to A,\text{ in time }dt) & =f\left(s_{i}(t)\right)\;dt,\\ P(A\to Q,\text{ in time }dt) & =\alpha \;dt, \end{array}$$

where $s_{i}(t)=\sum_{j}\frac{w_{ij}}{N}a_{j}(t)+h_{i}$ is the input to the neurone. Here, *f* is an activation function, $h_{i}$ is an optional external input, $w_{ij}$ is the connection strength from neurone *i* to neurone *j*, and $a_{j}(t)=1$ if neurone *j* is active at time *t* and zero otherwise. *α* is the de-activation rate and, therefore, controls the refractory period of the neurone.

To allow tractability, we further make the following simplifications: 

1. We assume that all synaptic weightings are equal ($w_{ij}=w$).

2. We assume there is no external input. The driven case will be explored theoretically and empirically in a companion manuscript.

3. We assume the linear identity activation function f(x)=x. Although it is more common to use sigmoid activation functions, we note that the identity function can just be thought of as a suitably scaled *tanh* function over the desired range.

As the network is fully connected, we can write the following mean field equation for active neurones:

$$\frac{dA}{dt}=\frac{wA}{N}Q-\alpha A=\frac{wA}{N}(N-A)-\alpha A,$$

where we have appealed to the fact that the system is closed, and thus A+Q=N. This ODE is analogous to the much studied [37] susceptible → infectious → susceptible (SIS) model used in mathematical epidemiology and we can appeal to some of the known results in studying its behaviour. Primarily, we can use simple stability analysis. The non-zero steady state is given by $A^{*}=N(1-\alpha /w)$. Setting $g(A)=\frac{dA}{dt}$, this equilibrium point is stable if $g^{\prime }(A^{*})<0$. Thus,

$$g^{\prime }(A)=(w-\alpha )-2w\frac{A}{N}\;\Rightarrow \;g^{\prime }\left(A^{*}\right)=(w-\alpha )-2w\frac{N(1-\alpha /w)}{N}=\alpha -w.$$

**Fig. 1:**
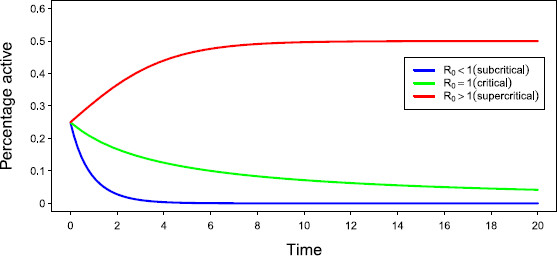
*Activity in the different regimes.* Plot of the solution to the ODE for N=800 and three different regimes where $R_{0}$ was set to 0.5 (*blue*), 1.0 (*green*) and 2.0 (*red*). Initially we activated 25 % of the network

Borrowing from epidemiology, we define the threshold $R_{0}=\frac{w}{\alpha }$. If $R_{0}>1$, we see that $g^{\prime }(A^{*})=\alpha -w<0$ and the non-zero steady state is stable. Figure 1 illustrates the differing behaviour of the solution to the above ODE for $R_{0}<1$ (sub-critical), $R_{0}=1$ (critical), and $R_{0}>1$ (super-critical). 

### 2.1 Firing Neurones and Avalanches

Instead of focussing on the average activity level across the network, we will instead look at the size distribution of firing neurones following one firing event. To do this, we begin with a fully quiescent network and initially activate just one neurone. We then record the total number of neurones that fire (the number of quiescent to active transitions) until the network returns to the fully quiescent state. We use this process of sequential activation as our definition of an avalanche and our main interest is the distribution of the avalanche sizes. Unfortunately, the simple ODE approach will not provide us with this distribution. To calculate this distribution, we use the semi-analytic approach described in the following section.

### 2.2 Tree Approach to the Avalanche Distribution

We begin by defining $q_{i}$ as the probability the next transition is a recovery (from A to Q) given *i* active neurones (i>0). The probability the next transition is an activation is then $1-q_{i}$ and we have:

$$\begin{array}{rl} q_{i} & =\frac{\alpha N}{w(N-i)+\alpha N}=\frac{N}{R_{0}(N-i)+N},\\ 1-q_{i} & =\frac{w(N-i)}{w(N-i)+\alpha N}=\frac{R_{0}(N-i)}{R_{0}(N-i)+N}. \end{array}$$

**Fig. 2:**
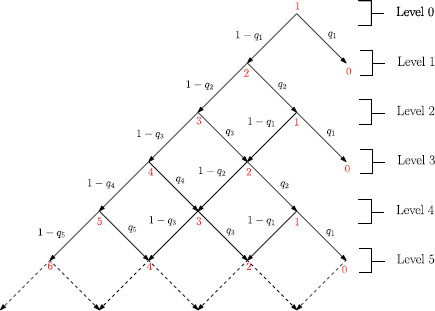
*First six levels of the probability tree.Red numbers* are the number of active neurones, *black values* are the probability of transitions between levels and sub levels

In order to calculate the avalanche size distribution, we adopt a recursive approach. We begin by considering the process unfolding in a tree-like manner with 1 initially active neurone. The tree can be divided into levels based on the number of transitions that have occurred and how the process is unfolding. Let level *j* contain the possible number of active neurones after *j* transitions. The recursive tree approach relates the probability of transition between levels to the final avalanche size. Figure 2 illustrates the initial levels of this process. 

To continue we define $p_{j}^{i}$ as the probability of having *i* active neurones on level *j* with i=0,1,2,…,N and $j\in N_{0}$. Assuming initially only one active neurone, we immediately see that $p_{0}^{1}=1$, $p_{1}^{2}=1-q_{1}$ and $p_{1}^{0}=q_{1}$. To proceed, we will consider the probability of having a particular number of active neurones on an arbitrary level. First, we note the following relation between levels:

$$p_{j}^{i}=\{\begin{array}{cc} p_{j-1}^{2}q_{2}, & \text{if }i=1,\\ p_{j-1}^{i-1}(1-q_{i-1})+p_{j-1}^{i+1}q_{i+1}, & \text{for }1<i<N,\\ p_{j-1}^{N-1}(1-q_{N-1}), & \text{if }i=N. \end{array}$$

We now define:

$$p(l)=\left(\begin{array}{c} p_{l}^{1}\\ \vdots \\ p_{l}^{N} \end{array}\right).$$

We can now write p(l+1)=A⋅p(l) where matrix **A** is given by the following tridiagonal matrix:

$$A=\left(\begin{array}{ccccccc} 0 & c_{1} & & & & & \\ \ddots & \ddots & \ddots & & & & \\ & \ddots & \ddots & \ddots & & & \\ & & b_{i} & 0 & c_{i} & & \\ & & & \ddots & \ddots & \ddots & \\ & & & & \ddots & \ddots & \ddots \\ & & & & & b_{N} & 0 \end{array}\right)$$

with $b_{i}=(1-q_{i-1})$ and $c_{i}=q_{i+1}$.

On the *j*th level of the tree, the probability of only 1 neurone being active is given by $p_{j}^{1}$. As on level 0, we began with only a single active neurone then for *j* odd, $p_{j}^{1}$ is always equal to zero. For *j* even, say j=2k, then as we began with only one active neurone on level 0, to have only one active neurone on level *j* means that *k* firings must have occurred. We can then calculate the probability of zero active neurones after *k* firings as $q_{1}p_{2k}^{1}$; this is thus the probability, P(k+1), of having an avalanche of size k+1 (or size *k* if we were not to include the initial active neurone). Setting $e={\left(1,0,0,\ldots ,0\right)}^{T}$ and noting that $p_{2k}^{1}=e^{T}A^{2k}e$ we have $P(k+1)=q_{1}e^{T}A^{2k}e$. To calculate the distribution, we implemented this recursive method of calculation in the MATLAB^®^ environment. Whilst this result is exact, and will be referred to as such henceforth, it can only be calculated numerically via recursion and cannot be given in the form of a closed expression.

### 2.3 Simulations of Neuronal Avalanches

In order to check the validity of our method, we performed simulations of the firing neurones using the Gillespie algorithm [38]. Due to the network being fully connected the algorithm is relatively straightforward: 

• As earlier, let *A* be the number of active neurones in the network (*Q* the number of quiescent). Given that an individual neurone becomes quiescent at rate *α* then the total rate of (Active → Quiescent) transitions is given by $r_{aq}=A\alpha$. Similarly, the total rate of (Quiescent → Active) transitions is given by $r_{qa}=f(s_{i})Q=f(s_{i})(N-A)$.

• Let $r=r_{aq}+r_{qa}$ and generate a timestep *dt* from an exponential distribution of rate *r*.

• Generate a random number *n* between 0 and 1. If $n<\frac{r_{aq}}{r}$ an active neurone turns quiescent, otherwise a quiescent neurone is activated (fires). This event is said to occur at time t+dt and the network is updated accordingly.

### 2.4 Exact Solution Compared to Simulation

**Fig. 3:**
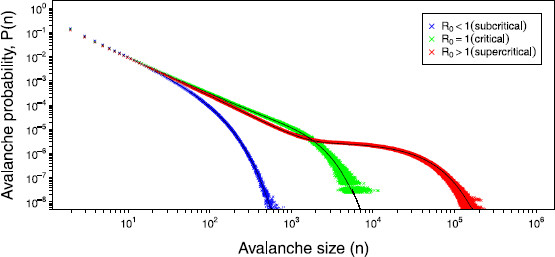
*Avalanche distributions.* Results from the simulations of the avalanche distributions for the subcritical ($R_{0}<1$, *blue*), critical ($R_{0}=1$, *green*) and supercritical ($R_{0}>1$, *red*) regimes for a network of size N=800. For each regime 2,000,000,000 avalanches were simulated. The corresponding exact solutions are shown in *black*

Values of the threshold, $R_{0}$, were chosen less than, equal to and finally above 1. We will refer to these regimes as subcritical, critical, and supercritical, respectively. Figure 3 illustrates the, as expected, good agreement between the simulations and the exact result for the three different regimes of $R_{0}$. 

### 2.5 Comparing the Exact Solution to a Closed Form Approximation

In [36], Kessler proposed a closed solution to the analogous susceptible-infected-susceptible (SIS) problem where he was interested in the number of infections (including reinfections) occurring over the course of an epidemic. For small avalanche sizes where the number of infectives is negligible compared to the network size, the transition probabilities can be approximated as

$$\begin{array}{rl} q_{i} & =\frac{N}{R_{0}(N-i)+N}\approx \frac{1}{R_{0}+1},\\ 1-q_{i} & =\frac{R_{0}(N-i)}{R_{0}(N-i)+N}\approx \frac{R_{0}}{R_{0}+1}. \end{array}$$

In the critical regime $R_{0}=1$, the problem reduces to calculating the distribution of first passage times of a random walk with equal transition probabilities. Thus, for avalanche sizes in the range, $1\ll n\ll \sqrt{N}$, Kessler [36] gave the following distribution based on Stirling’s approximation:

(1) P(n)=122n−1[(2n−2n−1)−(2n−2n)]≈14πn3.

We note however that the range over which the distribution can be shown to be a power law is rather limited and for small networks will not hold. Using the theory of random walks and a Fokker–Planck approximation, Kessler also derived the following closed solution to the probability distribution of infections in the critical regime ($R_{0}=1$) for larger sizes:

(2) $$P(n)=\frac{1}{\sqrt{4\pi N^{3}}}\exp(n/2N)\sinh^{-3/2}(n/N)\;(n\gg 1).$$

**Fig. 4:**
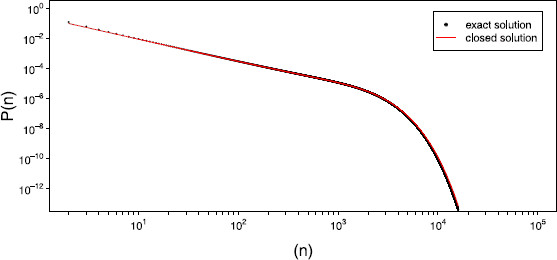
*Closed solution versus exact.* Plot of the closed solution (*red solid line*) versus the exact solution (*black dots*) for a network of size N=800 operating in the critical regime

Figure 4 plots this approximation against our exact solution for a network of size N=800. To more formally assess the convergence of the approximate solution to that of our exact solution, we considered the probabilities of avalanches from size N/10 to 20*N* and measured the difference between the distributions using two different metrics. Letting $P_{e}(n)$ be the exact probability of an avalanche of size *n* and $P_{k}(n)$ be the Kessler approximation to this, we first considered the standard mean square error given by

$$\mathrm{Error}(N)=\frac{1}{R}\sum_{n=N/10}^{20N}{\left(P_{e}(n)-P_{k}(n)\right)}^{2}\;\text{where }R=20N-N/10+1.$$

Secondly, we considered a more stringent measure of the error by looking at the supremum of difference between the same range of avalanches

$$\mathrm{Error}(N)=\underset{n}{\sup}\left|P_{e}(n)-P_{k}(n)\right|.$$

**Fig. 5:**
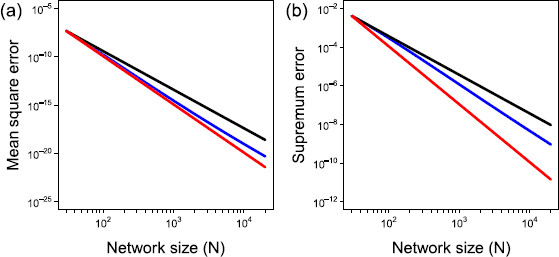
*Convergence of closed solution to exact.***a** Here the mean square error is given by the *blue line* and $O(N^{2})$ and $O(N^{3})$ convergence represented by the *black* and *red lines* respectively. **b** Here the supremum error is given by the *blue line* and $O(N^{4})$ and $O(N^{5})$ convergence represented by the *black* and *red lines* respectively

Figure 5 illustrates the two errors for increasing network size and both show how the proposed closed solution is indeed converging to that of the exact. 

## 3 Scale-Free Behaviour in the $R_{0}=1$ Regime

**Fig. 6:**
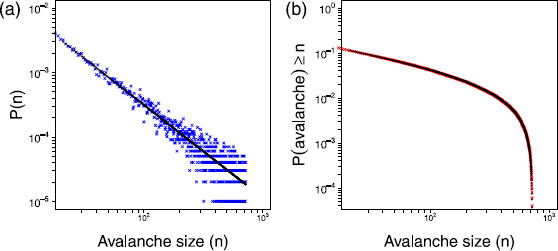
*Fitted distributions.* Out of 100,000 of the observed avalanches we fit the 98,833 whose size was less than $\frac{9}{10}N$. **a** The fitted probability distribution function (*black line*) fitted over the simulated avalanche distribution (*blue dots*). **b** The fitted cumulative distribution function (*black line*) fitted over the simulated avalanche distribution (*red dots*)

Whilst Eq. (1) gives a power law, this equation only holds over a limited range. Equation (2), in turn, is neither a power law nor a truncated power law. Here, we assess the range over which the distribution of sizes can be said to exhibit scale-free behaviour. For a rigorous assessment of this range, we employ a subset of the model selection approach described by Clauset and colleagues [24]. Specifically, we consider 100,000 of the simulated avalanches described earlier, and fit a truncated power law distribution of the form $P(x)=Cx^{-\alpha }$ to avalanches up to size $x_{\max}=\frac{9}{10}N$ (the choice of this upper bound will be justified in the following section) by using the maximum likelihood method (here *C* is a normalising constant to keep the sum of the distribution between $\left[x_{\min},x_{\max}\right]$ equal to 1). We do this by finding values of *α* and $x_{\min}$ that maximise the probability of obtaining our simulated avalanches given the fitted distribution. Next, we randomly generate 1,000 data sets from the fitted distribution and compute the difference between these synthetic data sets and the fitted form (using the Kolmogorov–Smirnov statistic). Similarly, we compute the difference between our simulations and the fitted power law. The *p*-value is then calculated as the proportion of synthetic data sets that are further away from the theoretical distributions than our simulations. As per [24], the hypothesis (that the data comes from a power law) is rejected if the *p*-value is less than 0.1. Note that in the model selection approach, should the hypothesis not be rejected, then one should test alternative models and use an information criterion to identify the best model. However, our focus here is purely on assessing whether our distribution can be said to behave like a power law distribution (we know it is not actually a power law) and therefore alternative models were not tested. With 100,000 avalanches, we obtained a *p*-value of 0.382 leading us not to reject the hypothesis that the distribution was power law (see Fig. 6). Since the distribution is not a power law, we would expect that upon considering a larger number of avalanches, this hypothesis should be rejected [23]. Indeed, using data from 1,000,000 avalanches yielded a *p*-value of 0, i.e., the truncated power law is not an appropriate model for the distribution. 

**Fig. 7:**
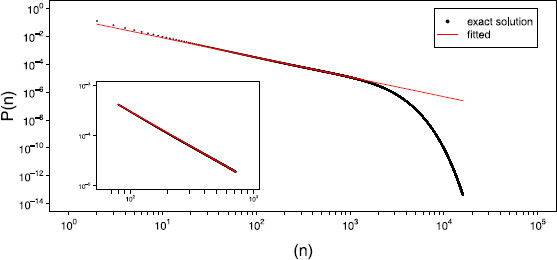
*Power law fit of the exact solution.Main*: plot of the truncated power law (*red line*) fitted over the entire range of the exact distribution (*black dots*). *Inset*: Fitted power law and exact distribution in the range [$\frac{1}{10}N,\frac{9}{10}N$]

The fact that the truncated power law was a plausible fit for the fewer number of avalanches (note that 100,000 is of the same order of magnitude as the number of avalanches typically reported in in vitro or in vivo studies of neuronal avalanches) is indicative of the partial scale-free behaviour the model exhibits. The appeal of the concept of critical brain is that the critical regime is the one in which long-range correlations keep the system poised between too highly correlated states of no behavioural value and too weakly correlated states that prevent information flow [39]. Thus, the actual nature of the distribution of the avalanche size matters less than any indication of the presence of long range correlations. In other words, neuronal avalanches need not precisely follow a power law, they just need to exhibit similar behaviour. It is important to appreciate this distinction. As the exact solution to the distribution of avalanche sizes is known, we can then compare it visually with a fit of a truncated power law over avalanche sizes from $\frac{1}{10}N$ to $\frac{9}{10}N$. This is done in Fig. 7, which confirms that over a limited range of sizes the distribution is well approximated by a power law. 

## 4 Origin of the Distribution’s Truncation

The fact that we have an exact form for the distribution allows us to make further important observations about some of its characteristics. Here, we explore the origin of the distribution’s truncation. Let $\lambda_{1},\lambda_{2},\ldots ,\lambda_{N}$ be the eigenvalues of **A** with the corresponding eigenvectors $u_{1},u_{2},\ldots ,u_{N}$. The initial condition can then be given as $p(0)=c_{1}u_{1}+c_{2}u_{2}+\cdots +c_{N}u_{N}$. As the matrix **A** is similar to a symmetric tridiagonal matrix with real entries (consider the diagonal similarity transformation matrix *D*, with $D_{1}=1$ and $D_{j}=\sqrt{\left(b_{j}b_{j-1}\cdots b_{2})/(c_{j-1}c_{j-2}\cdots c_{1}\right)}$), we know that its eigenvalues are real.

Using the property $Au_{j}=\lambda_{j}u_{j}$ we then obtain $p(k)=c_{1}\lambda_{1}^{k}u_{1}+c_{2}\lambda_{2}^{k}u_{2}+\cdots +c_{N}\lambda_{N}^{k}u_{N}$. This calculation leads to the probability of an avalanche being of size *n* being:

(3) $$P(n)=q_{1}\sum_{i=1}^{N}d_{i}\lambda_{i}^{2n},$$

where $q_{1}$ is the probability that the next transition is a recovery (from *A* to *Q*) given 1 active neurone (as defined earlier), $\lambda_{i}$ are the eigenvalues of the transition matrix **A** and $d_{i}$ are specified by the eigenvectors of the transition matrix and the initial conditions. We note that the earlier equation, $p(0)=c_{1}u_{1}+c_{1}u_{1}+\cdots +c_{N}u_{N}$, can be solved to obtain $c_{i}$. Using this, we can then calculate $d_{i}$ as the first entry of the vector $c_{i}u_{i}$. Equation (3), which is exact, thus demonstrates that the distribution of avalanche sizes is a linear combination of exponentials.

The structure of **A** (namely the all zero diagonal) means that if $u=(u_{1},u_{2},\ldots ,u_{N-1},u_{N})$ is an eigenvector with corresponding eigenvalue $\lambda_{u}$, then $v=(u_{1},-u_{2},\ldots ,{\left(-1\right)}^{N}u_{N-1},{\left(-1\right)}^{N+1}u_{N})$ is an independent eigenvector with corresponding eigenvalue $-\lambda_{u}$ (here, and in all that follows, we are assuming *N* is even; for *N* odd there is an additional zero eigenvalue). Setting $\tilde{N}=\frac{N}{2}$ and $e_{i}=d_{i}+d_{N-i+1}$ allows us to rewrite Eq. (3) as

(4) $$P(n)=q_{1}\sum_{i=1}^{\tilde{N}}e_{i}\lambda_{i}^{2n}.$$

Assuming the lead eigenvalue is denoted by $\lambda_{1}$, then for all *i*, $\lambda_{i}<\lambda_{1}$ and we have

$$\begin{array}{rl} \frac{P(n)}{q_{1}e_{1}\lambda_{1}^{2n}} & =\sum_{i=1}^{\tilde{N}}\frac{e_{i}\lambda_{i}^{2n}}{e_{1}\lambda_{1}^{2n}}\\ & =\frac{e_{1}}{e_{1}}{\left(\frac{\lambda_{1}}{\lambda_{1}}\right)}^{2n}+\frac{e_{2}}{e_{1}}{\left(\frac{\lambda_{2}}{\lambda_{1}}\right)}^{2n}+\cdots +\frac{e_{\tilde{N}}}{e_{1}}{\left(\frac{\lambda_{\tilde{N}}}{\lambda_{1}}\right)}^{2n}. \end{array}$$

Taking the limit as *n* increases, we find

$$\lim_{n\to \infty }\frac{P(n)}{q_{1}e_{1}\lambda_{1}^{2n}}=1.$$

**Fig. 8:**
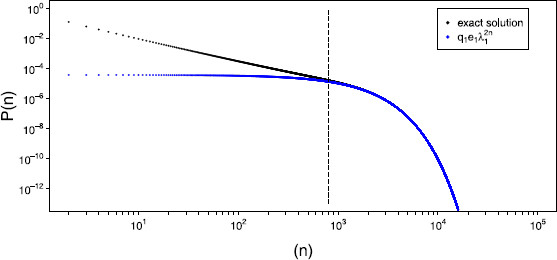
*Exponential cutoff.* Exact avalanche distribution (*black dots*), plotted against a distribution assuming only the leading eigenvalue is non-zero (*blue dots*). Avalanches greater than the system size, N=800, appear after the *dashed line*

Hence, $P(n)∼q_{1}e_{1}\lambda_{1}^{2n}$ and for larger avalanche sizes we have the leading eigenvalue dominating thus giving the exponential cutoff observed. We illustrate this convergence in Fig. 8 where we plot the exact avalanche distribution, P(n), against $q_{1}e_{1}\lambda_{1}^{2n}$. This figure also illustrates that the leading eigenvalue begins to dominate for avalanches just over the system size. It is for this reason that we chose an upper bound of $\frac{9N}{10}$ when fitting a power law to the distribution of avalanche sizes in the previous section. 

Such a distribution as (4) could converge to a true power law under two important conditions: 

1. the eigenvalues are well approximated by a geometric distribution, i.e., they are in the form $\lambda_{i}=Ke^{-(\mu /2)i}$,

2. the constants, $e_{i}$, are well approximated by $e_{i}=Li^{q}$,

 where *K*, *μ*, *L*, and *q* can be inferred via a numerical fitting procedure. In such a scenario, Eq. (4) can be rewritten to give

(5) $$P(n)=C\sum_{i=1}^{\tilde{N}}i^{q}{\left(e^{\mu n}\right)}^{-i},$$

where *C* is a given constant. In the limit of an infinite network size, we then have

(6) $$P(n)=C\sum_{i=1}^{\infty }i^{q}{\left(e^{\mu n}\right)}^{-i}.$$

While P(n) can be found based on standard mathematical arguments, we have chosen to use results derived in the context of the **Z**-transform. The standard results for integer values of *q* give

(7) $$\sum_{i=1}^{\infty }i^{q}z^{-i}={\left(-1\right)}^{q}D^{q}\left(\frac{z}{z-1}\right),$$

where *D* is an operator such that $D(f(z))=z\frac{d(f(z))}{dz}$. For a fixed integer value of *q*, an approximation for P(n) can be obtained by simply applying the operator as many times as necessary and then substituting $z=e^{\mu n}$. For q=1, for example, $P(n)\propto \frac{e^{\mu n}}{{\left(e^{\mu n}-1\right)}^{2}}$ which for small values of *μ* is well approximated by $\frac{1}{\mu^{2}}\frac{1}{n^{2}}$.

These results only hold for integer values of *q* so an alternative approach is to approximate the sum for P(n) in terms of an integral. Taking into account the special form for the eigenvalues and constants, P(n) can be approximated as follows:

(8) $$P(n)=C\sum_{i=1}^{\infty }i^{q}{\left(e^{\mu n}\right)}^{-i}≃C\int_{0}^{\infty }x^{q}e^{-\mu nx}\;dx.$$

The latter integral can be interpreted as a Laplace transform of $x^{q}$ , and thus yields

(9) $$P(n)≃C\frac{\Gamma (q+1)}{\mu^{q+1}}\frac{1}{n^{q+1}}.$$

It is worth noting that this result is consistent with that obtained for integer values of *q*.

**Fig. 9:**
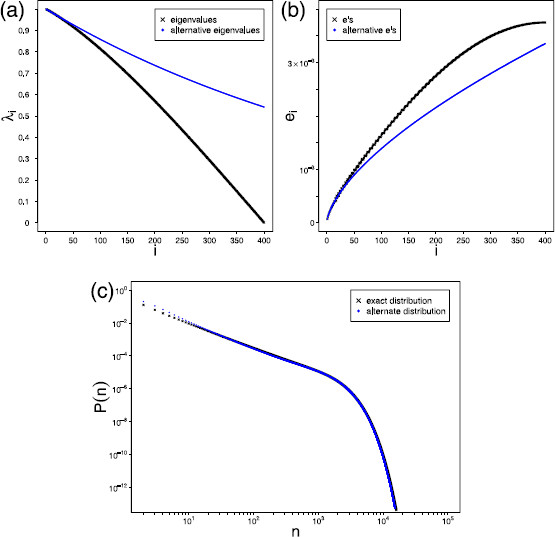
*Possible origin of the power law for large systems.***a** Actual distribution of eigenvalues $\lambda_{i}$ (*black crosses*) along with fitted distribution (*blue dots*). **b** Actual distribution of constants $e_{i}$ (*black crosses*) along with fitted distribution (*blue dots*). **c** Exact distribution of avalanche sizes (*black crosses*) along with distribution resulting from fitted distributions of $\lambda_{i}$ and $e_{i}$ (*blue dots*). All *plots* are for a network of size N=800 operating at criticality

For a simple empirical verification of this conjecture, we determined the values of *K*, *μ*, *L*, and *q* in the above conditions through numerical fitting of the first 23 eigenvalues and *e* constants of the exact distribution for a network of size N=800 (see Fig. 9(a), (b)) and compared the resulting probability distribution with the exact distribution. Whilst the lesser valued eigenvalues and larger e values were not fitted well, Fig. 9(c) shows there is still remarkable agreement between both curves over a broad range of values, including the range [$\frac{1}{10}N,\frac{9}{10}N$] over which a power law like behaviour was established earlier (see Fig. 7). This result clearly illustrates the dominance of the larger eigenvalues and, given that the fitted distribution converges to a power law, gives support to the conjecture that the exact distribution would do so in the limit of an infinite network. 

## 5 Other Markers of Criticality

Since the distribution of avalanche sizes in the finite-size critical system does not necessarily follow a true power law, the application of robust statistical testing in experimental conditions could well lead to rejecting the hypothesis that the data may come from a system operating in the critical regime. Therefore, in this section, we consider two experimentally testable markers of criticality: critical slowing down and divergence of susceptibility. We will define those concepts below but first we briefly summarise Van Kampen’s system size expansion [40], which we use to illustrate those markers on our system. 

### 5.1 System Size Expansion

For generality, we now assume that each neurone receives a constant external input and that the activation function can take forms other than the simple identity function. We define the probability that the number of neurones active at time *t* is *n* as $P_{n}(t)$. Then the master equation can be given as

$$\begin{array}{rl} \frac{dP_{n}(t)}{dt}= & \alpha (n+1)P_{n+1}(t)\\ & -\alpha nP_{n}(t)\\ & +f\left(\frac{w(n-1)}{N}+h\right)\left(N-(n-1)\right)P_{n-1}(t)\\ & -f\left(\frac{wn}{N}+h\right)(N-n)P_{n}(t). \end{array}$$

The idea of the system size expansion is to now model the number of active neurones as the sum of a deterministic component scaled by *N* and a stochastic perturbation scaled by $\sqrt{N}$, i.e.,

$$n(t)=N\mu (t)+N^{1/2}\xi (t).$$

A more detailed explanation of this can be found in [12] and [40], but importantly what is obtained is the following set of equations for *μ* (which is the solution to the mean field equation of the proportion of active neurones), 〈ξ〉 (the expected value of the fluctuations) and $\sigma^{2}=\langle \xi^{2}\rangle -{\langle \xi \rangle }^{2}$ (the variance of the fluctuations)

(10) $$\frac{\partial \mu }{\partial t}=-\alpha \mu +(1-\mu )\hat{f},$$

(11) $$\frac{\partial \langle \xi \rangle }{\partial t}=-\left(\alpha +\hat{f}-w{\hat{f}}^{\prime }(1-\mu )\right)\langle \xi \rangle ,$$

(12) $$\frac{\partial \langle \sigma^{2}\rangle }{\partial t}=-2\left(\alpha +\hat{f}-w{\hat{f}}^{\prime }(1-\mu )\right)\langle \sigma^{2}\rangle +\left(\alpha \mu +(1-\mu )\hat{f}\right).$$

Here, $\hat{f}=f(w\mu +h)$ and ${\hat{f}}^{\prime }=f^{\prime }(w\mu +h)$. These equations, in turn, give the following equations for the mean, *A*, and variance, $A_{\sigma }$, of the number of active neurones:

(13) $$\begin{array}{rl} A & =N\mu +N^{-1/2}\langle \xi \rangle \\ & =N\mu \;\text{(assuming we know the initial number of active neurones)}, \end{array}$$

(14) $$A_{\sigma }=N\langle \sigma^{2}\rangle .$$

We make use of these equations in the following two sections.

#### 5.1.1 Critical Slowing Down

In dynamical systems theory, a number of bifurcations, including the transcritical bifurcation in our system, involve the dominant eigenvalue characterising the rates of changes around the equilibrium crossing zero. As a consequence, the characteristic return time to the equilibrium following a perturbation increases when the threshold is approached [41]. This increases has led to the notion of critical slowing down as a marker of critical transitions [42]. Here, we illustrate the critical slowing down of our model with the analytic derivation of the rate of convergence to the steady state (this derivation has been previously shown by [43]). We first begin by calculating the analytic solution to Eq. (10) for the percentage of active neurones. We again consider the case where *f* is the identity function and can thus write

(15) $$\frac{\partial \mu }{\partial t}=-\alpha \mu +(1-\mu )f(w\mu +h)=-\alpha \mu +(1-\mu )(w\mu +h).$$

Assuming zero external input (h=0), we have

(16) $$\frac{\partial \mu }{\partial t}=-\alpha \mu +(1-\mu )(w\mu +h)=-\alpha \mu +(1-\mu )w\mu .$$

We are interested in the solution of this equation and consider the result for different values of *α*. Firstly, we consider α≠w. In this case, we have

(17) $$\frac{\partial \mu }{\partial t}=-\alpha \mu +(1-\mu )w\mu =\mu (w-w\mu -\alpha ).$$

Integrating this using separation of variables and the initial condition $\mu (0)=\mu_{0}$, we find

(18) $$\mu (t)=\frac{w-\alpha }{Ae^{(\alpha -w)t}+w}\;\text{where }A=\frac{\mu_{0}}{w-w\mu_{0}-\alpha }.$$

The solution to this depends on whether α<w or α>w ($R_{0}>1$ and $R_{0}<1$, respectively). If α<w, then as t→∞, $\mu \to \frac{w-\alpha }{w}$. If α>w then as t→∞, μ→0. Note that in both cases, convergence of the number of active neurones to the steady state solution is exponential.

Now we consider the solution when α=w, i.e., the critical regime

$$\frac{\partial \mu }{\partial t}=-\alpha \mu +(1-\mu )\alpha \mu =-\alpha \mu^{2}\Rightarrow \mu (t)=\frac{1}{\alpha t+\mu_{0}^{-1}}.$$

Thus, as t→∞ we find μ(t)→0. However, unlike for $R_{0}\neq 1$, convergence to the steady state exhibits a power law dependence on time [43]. 

#### 5.1.2 Divergence of Susceptibility

A correlate of the phenomenon of critical slowing down is that of the divergence of susceptibility of the system as the system approaches the bifurcation [42]. In this section, we investigate the behaviour of the equation for the variance. For simplicity, we consider again the case of the identity activation function and a non-driven system h=0. First, we use Eq. (12) to calculate the variance in the percentage of active neurones:

$$\begin{array}{rl} \frac{\partial \sigma^{2}}{\partial t} & =-2\left(\alpha +\hat{f}-w{\hat{f}}^{\prime }(1-\mu )\right)\sigma^{2}+\left(\alpha \mu +(1-\mu )\hat{f}\right)\\ & =-2\left(\alpha +w\mu +h-w^{2}(1-\mu )\right)\sigma^{2}+\left(\alpha \mu +(1-\mu )(w\mu +h)\right)\\ & =-2\left(\alpha +w\mu -w^{2}(1-\mu )\right)\sigma^{2}+\left(\alpha \mu +(1-\mu )w\mu \right). \end{array}$$

Setting this equal to zero and rearranging, we obtain

$$\sigma^{2}=\frac{\left(\alpha \mu +(1-\mu )w\mu \right)}{2(\alpha +w\mu -w^{2}(1-\mu ))}=\frac{\left(\mu +(1-\mu )R_{0}\mu \right)}{2(1+R_{0}\mu -R_{0}w(1-\mu ))}.$$

Here, we note that unlike the equation for *μ* where there was only the single bifurcation parameter $R_{0}$, we now have the additional dependence on *w*. To maintain consistency with earlier results, we now set w=1 to obtain

$$\lim_{t\to \infty }\sigma^{2}(t)=\{\begin{array}{cc} \alpha & \text{if }\alpha <1\;(R_{0}>1),\\ \frac{1}{2} & \text{if }\alpha =1\;(R_{0}=1),\\ 0 & \text{otherwise }(R_{0}<1). \end{array}$$

Using Eq. (14), we obtain

$$\lim_{t\to \infty }{\langle A\rangle }_{\sigma }=\lim_{t\to \infty }N\langle \sigma^{2}\rangle =\{\begin{array}{cc} \frac{N}{R_{0}} & \text{if }R_{0}>1,\\ \frac{N}{2} & \text{if }R_{0}=1,\\ 0 & \text{otherwise }(R_{0}<1). \end{array}$$

**Fig. 10:**
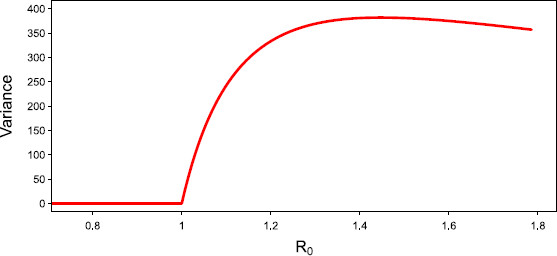
*Divergence of susceptibility.* Analytic result for the steady state of the variance as $R_{0}$ approaches 1 in a network of size N=800. Results only provided down to α=2/3 for clarity

Figure 10 illustrates the jump to a non-zero steady state when the critical value $R_{0}=1$ is approached from below, and the divergence in variance when it is approached from above. 

Here, it should be noted that any finite-size network has a zero absorbing state so that eventually all activity will die out irrespective of the value of $R_{0}$. However, it has been shown that the ODE limit is a valid approximation to the solution of the master equation for reasonably sized systems with values of $R_{0}$ greater than 1 and only over a finite time horizon (see [44] for further discussion). Defining the true (i.e., calculated directly from the master equation for P(n)) expected value of active neurones at time *t* as $\tilde{A}(t)$, the convergence of the ODE approximation for A(t) given by Eq. (13) is such that for any t≥0$\lim_{N\to \infty }|A(t)-\tilde{A}(t)|=0$[45]. 

## 6 Discussion

Over the last decade or so, the search for evidence that the brain may be a critical system has been the focus of much research. This is because it is thought that a critical brain would benefit from maximised dynamic range of processing, fidelity of information transmission and information capacity [46]. Whilst support for the critical brain hypothesis has emerged from comparing brain dynamics at various scales with the dynamics of physical systems at criticality (e.g., [31,34,47-50]), in this paper, we focus on the important body of work that has relied on characterising power laws in the distributions of size of neuronal avalanches [8,30]. Our focus on this scale is motivated by empirical considerations regarding how one can go about demonstrating the above functional properties. Shew and Plenz [46] remark that any research strategy to test whether these properties are optimal near criticality will have to achieve two criteria: a means of altering the overall balance of interactions between neurones and a means of assessing how close to criticality the cortex is operating. As argued by these authors, the study of neuronal avalanches offers the greatest likelihood of achieving those two criteria. 

The importance of a robust assessment of the statistical properties of the avalanche size is therefore two-fold: on the one hand, it is about ascertaining the extent to which the system being studied has the statistical properties expected of a system operating at, or near, criticality; on the other hand, it is about being able to confirm that a manipulation/perturbation of the system aimed to push the system away from this critical regime has been effective. This consideration therefore puts a lot of importance on the description of the statistics one should expect in such a system. In the current literature, the assumption of the distribution of avalanche sizes taking a power law functional form relies on an analogy between the propagation of spikes in a neuronal network and models of percolation dynamics or branching processes for which exact power laws have been demonstrated *in the limit of system size*. As a result of the importance of having a robust assessment of the expected presence of a power law, greater emphasis has recently been put on using a sound statistical testing framework, e.g., [24]. Whilst we are unaware of any study in which the criticality hypothesis was rejected due to failure of rigorous statistical testing (which we suspect is due to the necessarily small number of observations, as we will argue below), there is clear evidence that many authors are now using the methods of Clauset et al. [24] to confirm the criticality of their experimental findings, e.g., [12,23,29]. As a result, we feel that it is all the more important to confirm that the assumed power law functional form is indeed a sensible representation of what one should expect in in vivo and in vitro recordings, which, unlike the physical systems considered when deriving the power law statistics, are finite-size systems. The aim of the paper was therefore to consider a model of neuronal dynamics that would be simple enough to allow the derivation of analytical or semi-analytical results whilst (i) giving us a handle on the parameter controlling the fundamental principle thought to underlie criticality in the brain, namely, the *balancing* between processes that enhance and suppress activity (note that we are intentionally not referring to excitation and/or inhibition—we will return to this below) and (ii) allowing us to determine its distribution of avalanche sizes when operating in the critical regime. Note that because we are using a finite-size system, we are appealing to a normal form of standard bifurcation, here, a transcritical bifurcation, because it embodies all that needs to be known about the ‘critical’ transition (Sornette, private communication).

Our semi-analytic derivation of the true distribution of avalanche sizes in a finite-size system suggests that, even though it is approximately scale free over a limited range, the distribution is not a true power law. First, this has important implications for the interpretation of results from a robust statistical assessment of the distribution. Indeed, as has been discussed by Klaus and Plenz [23], with a large number of samples, any distribution that deviates from the expected distribution by more than noise due to sampling, will eventually yield a *p*-value such that the power law hypothesis will be rejected, thus leading to the potentially incorrect conclusion that the system is not critical. This is the case in our scenario where using 10^6^ avalanches lead to a rejection of the criticality hypothesis even though the system is tuned to the critical regime. In contrast, with 10^5^ avalanches (which is consistent with empirical observations), a *p*-value above threshold leads to not rejecting the hypothesis that the distribution is a power law even though we established it is not one.^a^ This finding therefore provides an important counterpart to the analytical results of Touboul and colleagues [29] who showed that thresholded stochastic processes could generically yield apparent power laws that only stringent statistical testing will reject. Whilst the stringent testing will reject the hypothesis of criticality for a system that is not necessarily critical, it may also reject the hypothesis of criticality for a system that is critical only because the actual distribution is not actually a power law. This ambiguity of the avalanche distribution in the finite-size system therefore requires that one should carefully consider to what fundamental property the idea of a critical brain actually appeals to. We suggest that the key appeal is that the brain can exhibit long-range correlations between neurones without it ever experiencing an over saturation of activity or long periods of inactivity. It then follows that the importance is not in the exact distribution obtained but in the approximately scale-free behaviour it exhibits. In turn, this highlights the importance of looking at other markers of criticality (which we will discuss below). 

Another important result of this work is to provide the beginning of a mechanistic explanation for an often alluded to (e.g., [51]) but never properly treated (as far as we are aware) observation that whereas avalanches in a critical system with re-entrant connections could in principle be arbitrarily long, and certainly, exceeding the number of recording sites, neuronal avalanches in in vitro or in vivo systems (and many computational models of self-organised criticality) often show a cut-off at the number of sites. Our work suggests that the lead eigenvalue of the transition matrix between states fully determine the location of this cut-off, which turns out indeed to be at about the system size, even if avalanches of up to 20 times the system size can be observed. This finding therefore provides some justification for setting, or accepting, a bound within which to apply a Clauset-type methodology (we note that various reports use different ranges, e.g., 80 % of system size in [17], roughly system size in [51]). It is worth remembering that the number of recording sites can have profound implications on the nature of the distribution observed [21]. 

In addition to providing results on the distribution of avalanche sizes, we also sought to explore other potential markers of criticality. We provided results on two other markers of criticality—critical slowing down and divergence of susceptibility—both of which again follow from a dynamical systems appreciation of a critical bifurcation, i.e., the behaviour of a system whose lead eigenvalue crosses zero. The appeal of those markers, which have been documented in many other natural processes, e.g., [42,52], but seldom at the mesoscopic brain level^b^ (see [53] for a rare example) is that (a) they strengthen the assessment of the system being critical and (b) may contribute to achieving the second criterion of Shew and Plenz [46]. Although the authors are not in a position to provide explicit recommendations for an experimental design, we believe that these markers are amenable to robust experimentation, e.g., through pharmacological manipulation. 

Whilst we hope we have convinced the reader of the potential importance of these findings, we also need to recognise that the very simplicity that makes analytical work possible does also raise questions regarding how physiologically plausible such a model is and, therefore, whether its conclusions should be expected to hold. Below, we address a few of the points worthy of further consideration.

### 6.1 Validity of Inferring Criticality in a Finite Network

**Fig. 11:**
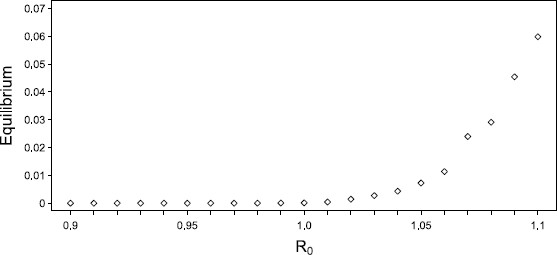
*Steady state versus*$R_{0}$. Plot of the steady state (averaged over 500 simulations at time t=150) obtained at $R_{0}$ values around the putative critical value of 1

In using the meanfield equations, it is important to understand how well they capture the behaviour and bifurcation structure of the stochastic process they are approximating. Whilst it is known that on the complete graph (see [54] for instance) and in the limit N→∞ the steady state solution of the ODE will converge to the expected value of the comparable stochastic process, it is unclear whether the critical point of the infinite system corresponds to that in the finite system. Furthermore, it is unclear whether a finite system can truly have a critical point and we must be cautious in claiming one exists. Importantly, however, it has been shown in [55] that for a complete graph, $R_{0}\approx 1$ (the paper proves the result for *α* fixed as 1 but the result is generalisable for any *α*) is the threshold below which the disease will die out quickly (expected time to extinction O(log(n))), and above which it dies out slowly (expected time to extinction $O(n^{a})$ for some *a*). Simulating the steady state of the network for increasing $R_{0}$ also shows (see Fig. 11) the characteristic feature of a second-order phase transition found at a critical point. For these reasons, whilst acknowledging the problem of inferring criticality in a finite regime, we feel justified in claiming $R_{0}=1$ as the critical point for the process unfolding on our finite network. 

### 6.2 Validity of a Purely Excitatory Network

In this paper, we have used a purely excitatory neuronal model. This not only simplifies the system but is also an important characteristic of the brain during early development. Experimental results have shown that during early development, before birth, GABAergic neurones (i.e., neurones which will later be inhibitory) have a depolarising effect on their post-synaptic neighbours [56-58]. Thus, our model might be considered as representative of early development. Power law statistics have been observed in early development at a time when networks are thought to be purely excitatory [32,59]. It should be noted that this approach has the benefit of casting a new light on the question of what is the minimum requirement for a neuronal system to show criticality. To a large extent, the current literature has been focused on a form of homeostasis resulting from either a fine balance between excitation and inhibition, e.g., [12,13] or some relatively complex dynamical processes at synaptic level, e.g., [17]. Our results show that a purely excitatory system can show the exact same behaviour such that on average each active neurone only activates one postsynaptic neurone. Here, this balanced state is achieved through a trade-off between the rates at which neurones become active and quiescent. It should be noted that this formulation of the problem leads to interesting parallels with classical models of mathematical epidemiology, which the authors intend to continue exploring. 

### 6.3 Spatial Structure

To make use of the analytic tractability of the mean field equation it was necessary to consider a fully connected network. While this is not true of the whole brain, it *may* be closer to the reality of the kind of in vitro systems typically considered in studies of neuronal avalanches. For example, Hellwig et al. [60] report up to 80 % connection probability in local connectivity between pyramidal neurones in layers 2/3 of the rat visual cortex. Extending the work presented here to consider the effect of network topology on the system’s dynamics and the resulting distribution of event sizes would be of particular interest from a developmental viewpoint (see, for instance, Larremore et al. [61], who have considered the avalanche distribution of general tree-like networks with discrete dynamics). As networks mature, there is not only a switch to inhibition by a proportion of the neurones (the so-called GABA switch), but also a subsequent pruning of synaptic connections [62]. The level of pruning is high, with a 40 % reduction in the number of synaptic connections between early childhood and adulthood [62]. Thus, a developing network may be more readily approximated by a fully connected network than an adult neural network would be. 

The lack of a spatial embedding of our model is in contrast with many classical models of criticality, and also with physiological systems. Accordingly, our model cannot display another important marker of criticality, namely, the divergence of correlation lengths in space. A spatial embedding is not needed for our system to be critical and to exhibit a distribution of avalanche size similar to that observed in physiological neuronal avalanches. It therefore begs the question of the exact role of spatial embedding in the dynamics of neuronal avalanches. It may well be that, just like balanced activity in our model comes about from a trade-off between excitation and refractoriness rather than between excitation and inhibition, specific spatial embeddings may enable balanced activity without the need for plastic mechanisms. Kaiser and Hilgetag [63] showed that hierarchical modular networks can lead to limited sustained activity whereby the activity of neural populations in the network persists between the extremes of either quickly dying out or activating the whole network. Roxin and colleagues [64] observed self-sustained activity in excitable integrate-and-fire neurones in a small-world network, whose dynamics depends sensitively on the propagation velocity of the excitation. 

### 6.4 Non-driven Case

Finally, in this paper, we have focused on the non-driven case h=0. Whilst this constraint allowed the derivation of analytical results, it obviously contrasts with the reality of a physiological system unless one considers that any ‘external’ input operates at such a slower timescale that one could assume separation of time scales (an important assumption in the self-organised criticality framework). However, the fact that binning is required for identifying avalanches in physiological recordings suggests that this separation of time scales is unlikely. Whilst the introduction of a non-zero *h* in our model does not affect the results obtained using finite size expansion, it does effectively make it impossible for the system to operate at $R_{0}=1$. A thorough investigation of the driven case (h>0) will be the subject of the companion paper.

## Competing Interests

The authors declare that they have no competing interests.

## Authors’ Contributions

TT carried out analysis and numerical simulations for tree approach, finite size expansion, critical slowing, comparison to Kessler’s approximate solution. TT and LB wrote the manuscript. CH carried out additional calculations and numerical simulations. PS and IK contributed the tree approach, and the derivation of the power law in the limit of the system size. LB conceived of the analysis and of the overall goals of the study, participated in the implementation and analysis of the different simulations. All authors read and approved the final manuscript.

## References

[Fn1] As the power law is not a sufficient condition of criticality, one should not infer from this that the system is indeed critical, however, this step is commonly taken in published reports and that is worth mentioning here.

[Fn2] Strictly speaking the notion of critical slowing in neurones firing near firing threshold appeals to the same notion.

